# Checkpoint Blocked; Lungs Impacted: A Case Report on Durvalumab-Induced Pneumonitis

**DOI:** 10.51894/001c.144642

**Published:** 2025-09-30

**Authors:** Sabita Shah

**Affiliations:** 1 Department of Internal Medicine Detroit Medical Center-Sinai Grace Hospital

## 121

### BACKGROUND

Durvalumab is an immune checkpoint inhibitor that targets PD-L1 and is used to treat various cancers, including non-small cell lung cancer. One potential side effect of immune checkpoint inhibitors like durvalumab is immune-related adverse events, including pneumonitis.

### CASE PRESENTATION

A 67-year-old female was diagnosed with stage I non-small cell lung cancer 3 years back and was treated with left upper lobe resection. Two years later, she experienced hemoptysis, leading to the discovery of invasive, poorly differentiated squamous cell carcinoma in the right lower lobe lung mass. She underwent endobronchial ultrasound and later received chemoradiation. A PET scan after three months showed some improvement. She began maintenance durvalumab but had to pause the third cycle two months later due to concerns about pneumonitis.

Two weeks later, she presented to the emergency department with worsening dyspnea and cough. Her initial vitals were remarkable for tachypnea and tachycardia. The physical exam was notable for expiratory wheezing. Laboratory findings showed mild leukocytosis, normocytic anemia, and hyperglycemia. CT thorax showed multifocal bilateral pneumonitis. The patient was transitioned to BiPAP. Broad-spectrum antibiotics and steroids were started. The patient continued to require a high dose of oxygen and was ultimately intubated on the 10th day of admission, given severe respiratory distress. An oncologist agreed to continue tapering dose steroids and recommended against intravenous immunoglobulin. She failed spontaneous breathing trial and ultimately had a tracheostomy and peg tube placed and discharged to long-term acute care facility.

### DISCUSSION/CONCLUSIONS

In the case of immune checkpoint inhibitors, such as durvalumab, pneumonitis is believed to occur due to the immune system’s overactivation. The mechanism behind durvalumab-induced pneumonitis likely involves the inhibition of the PD-1/PD-L1 pathway. When this pathway is blocked, the immune system may attack normal tissues, including the lungs, leading to inflammation and damage. Diagnosis is typically clinical, supported by imaging and sometimes biopsy. Management includes immediate discontinuation of durvalumab. The standard of care is high-dose steroids, which should be tapered over several weeks. In mild cases, patients may recover after discontinuing the drug and corticosteroid treatment. However, more severe cases can lead to permanent lung damage or death.

